# Synthesis, Cytotoxicity, and Antileishmanial Activity of N,N'-Disubstituted Ethylenediamine and Imidazolidine Derivatives

**DOI:** 10.1100/tsw.2010.176

**Published:** 2010-09-01

**Authors:** Gustavo S. G. de Carvalho, Patrícia A. Machado, Daniela T. S. de Paula, Elaine S. Coimbra, Adilson D. da Silva

**Affiliations:** ^1^ Departamento de Química, I.C.E., Universidade Federal de Juiz de Fora, Cidade Universitária, Juiz de Fora, Minas Gerais, Brazil; ^2^ Departamento de Parasitologia, Microbiologia e Imunologia, I.C.B., Universidade Federal de Juiz de Fora, Cidade Universitária, Juiz de Fora, Minas Gerais, Brazil

**Keywords:** imidazolidines, ethylenediamines, antileishmanial activity, Leishmania

## Abstract

This paper describes the preparation of N,N'-disubstituted ethylenediamine and imidazolidine derivatives and their in vitro biological activities against *Leishmania* species. Of the nine synthesized compounds, five displayed a good activity in both *L. amazonensis* and *L. major* promastigotes. The compounds 1,2-Bis(p-methoxybenzyl)ethylenediamine (4) and 1,3-Bis(p-methoxybenzyl)imidazolidines (5) showed the best activity on intracellular amastigotes, with IC_50_ values of 2.0 and 9.4 μ/mL, respectively. In addition, none of compounds were cytotoxic against mammalian cells. The leishmanicidal activity can be related with inhibition of polyamine synthesis and cellular penetration within biological membranes.

### INTRODUCTION

Leishmaniasis is a parasitic disease endemic in some tropical areas of the world and in underdeveloped countries, with an estimated 1.5 to 2 million cases per year[1]. It causes an estimated 70,000 deaths annually[1,2]. Chemotherapy against leishmaniasis in humans has been based on the pentavalent antimonials, such as sodium stibogluconate (Pentostam®) and meglumine antimoniate (Glucantime®)[1,2,3,4]. These drugs induce toxic side effects that require lengthy treatments with parental administration and increasing resistance[3]. The second-line compounds used in unresponsive cases include pentamidine and amphotericin B, but these drugs are very toxic[1,2,3,4].

An interesting chemotherapeutic approach in the design of novel antiparasitic drugs is the inhibition of parasitic biosynthesis of polyamines[5,6,7]. The hydrophobic moiety chains could interact with membrane lipids, facilitating the penetration of the drug into the cytoplasm where it interferes with the polyamine metabolism pathway of the parasite[5,6,7,8]. Additionally, due to the hydrophobic nature of

*These authors contributed equally in this work.
**Corresponding author.
©2010 with author.
Published by TheScientificWorld; www.thescientificworld.com imidazolidines, they were employed as ethylenediamine carriers. Recently, various derivatives of polyamines with antiparasitic activity have been introduced[9,10,11,12].

Imidazolidines, cyclic aminals of pharmacological interest, as well as N,N'-dibenzyl-2-arylimidazolidines, N,N'-bisaminoalkylimidazolidines, and N,N-dihydroxyphenylimidazolidines have shown fungicidal, antiparasitic, antibacterial, antiamebic, and antiviral activities[13,14]. Encouraged by this observation, the present paper describes the preparation of N,N'-disubstituted ethylenediamine and imidazolidine derivatives (Scheme 1) and their *in vitro* biological activities against *Leishmania* promastigote and amastigote forms. These types of compounds can be considered able to interact with membrane lipids, to be transported into the cytoplasm, and, possibly, to interfere with the lipid or polyamine transport or metabolism of the parasite[5,6,15,16,17].

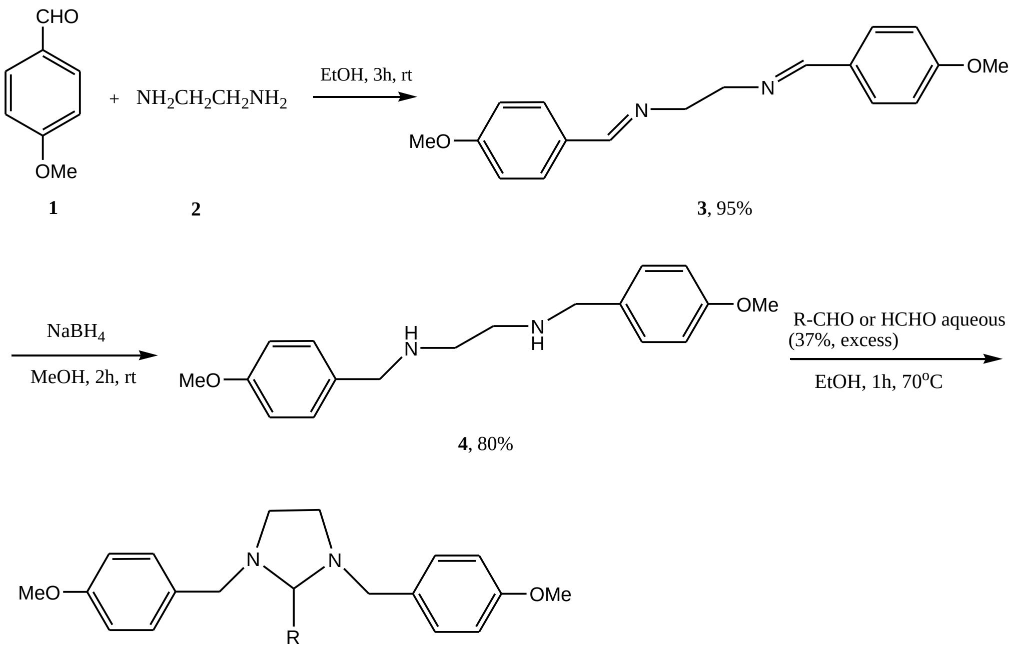

**5**: R= H
**6**: R= Ph
**7**: R= 4'-metoxiphenyl
**8**: R= 4'-hydroxy-5'-methoxyphenyl
**9**: R= 4'-dimethylaminophenyl
**10**: R= 4'-nitrophenyl
**11**: R= 2'-hydroxyphenyl

**5-11**, 70-80%

**SCHEME 1.** Reagents and conditions: (a) EtOH, 3h, rt; (b) $NaBH_4$, MeOH, 2h, rt; (c) aldehydes or aqueous formaldehyde (37%, excess), EtOH, 1h, 70ºC.

### MATERIALS AND METHODS

#### Chemicals

The imidazolidine derivatives **5–11** were synthesized by the classical method involving condensation between N,N'-disubstituted ethylenediamine **4** with a variety of aromatic aldehydes in EtOH (Scheme 1). N,N'-disubstituted ethylenediamine precursors were prepared following procedures in the literature. Those having benzyl substituents were synthesized by condensation of ethylenediamine **2** with aromatic aldehydes **1,** described previously[19], and further reduction of the generated Schiff bases with sodium borohydride, 70–80% yield[20]. All compounds were characterized by melting point (m.p.), $^{1}H$ and $^{13}C$ NMR (Table 1), and were in accordance with data in the literature[13,14,18].

**TABLE 1**
**Spectral Dates of Imidazolidine Derivatives**

| Compound | δ C*H*-(N)$_2$ | δ *C*-(N)$_2$ | M.P. (ºC) | Yield (%) |
|---|---|---|---|---|
| **5** | 5.39 (s, 2H) | 75.8 | Semisolid | 76 |
| **6** | 3.72 (s, 1H) | 89.1 | 92.0 | 74 |
| **7** | 3.68 (s, 1H) | 88.6 | 75.0–77.2 | 70 |
| **8** | 3.70 (s, 1H) | 88.9 | 104.7–106.5 | 75 |
| **9** | 3.75 (s, 1H) | 88.9 | 111.4–112.8 | 76 |
| **10** | 3.94 (s, 1H) | 87.7 | 150.3–150.7 | 74 |
| **11** | 3.93 (s, 1H) | 88.9 | 121.9–133.3 | 79 |

The experiments were performed at 300 MHz for $^1$H and 75 MHz for $^{13}$C in $CDCl_3$ and TMS as internal reference (δ 0.00 ppm).

### Biological Assays

Stock solutions of compounds were prepared at a concentration of 120 mg/mL in dimethyl sulfoxide (DMSO) and were kept frozen at –20$^o$C. Amphotericin B was supplied by Cristália (São Paulo, Brazil), prepared as a 10-mg/mL stock solution in Millipore water. Working solutions of compounds were prepared fresh for each use, by serial dilution of the stock solutions in Millipore water. Fetal bovine serum (FBS) was purchased from Cultilab (Campinas, São Paulo, Brazil). Brain heart infusion (BHI) was purchased from Himédia (Mumbai, India). Hemin, folic acid, RPMI 1640 medium, 3-(4,5-dimethylthiazol-2-yl)-2,5-diphenyl-tetrazolium bromide (MTT), and DMSO were purchased from Sigma Chemical (St. Louis, MO).

### *In vitro* Antileishmanial Activity

- Promastigote forms — For this assay, promastigotes of *L. amazonensis* (IFLA/Br/67/PH8) and *L. major* (MRHO/SU/59/P) were used. Promastigotes of *L. amazonensis* were cultured in Warren's medium (BHI, plus hemin and folic acid)[20] and promastigotes of *L. major* were maintained in medium BHI[21], both supplemented with 10% inactivated FBS at 24ºC. Log phase promastigotes of two *Leishmania* species were seeded in 96-well tissue culture plates ($2 \times 10^5$ cells/well). The parasites were exposed to increased concentrations of the compounds (at minimum six serial dilutions) for 72 h at 24°C and their viability was evaluated using a MTT assay, as described previously[22]. Amphotericin B was used as positive control. Controls containing 0.5% DMSO and medium alone were also included. The results are expressed as the concentrations inhibiting parasite growth by 50% ($IC_{50}$).
- Amastigote forms — Concerning the amastigotes *in vitro* model, macrophages were obtained from BALB/c mice previously inoculated with 3% thioglycolate medium[23]. Briefly, peritoneal macrophages were plated at $2 \times 10^5$ cells per well on coverslips (13-mm diameter) previously arranged in a 24-well plate in RPMI 1640 medium supplemented with 10% inactivated FBS and allowed to adhere for 24 h at 37°C in 5% $CO_2$. Adherent macrophages were infected with *L. amazonensis* (IFLA/Br/67/PH8) promastigotes in the stationary growth phase using a ratio of 1:5 at 37°C for 3 h. Noninternalized promastigotes were eliminated and solutions of tested compounds were added (nonserial five dilutions: from 45 to 1.5 µg/mL for compounds **4** and **5;** from 60 to 2.0 µg/mL for compounds **6**, **7,** and **11**). Then, the cells were maintained at 37ºC in 5% $CO_2$ for 72 h, fixed, and stained with Giemsa for parasite counting (optical microscopy,

1000× magnification). The survival index was obtained by multiplying the percentage of infected macrophages by the mean number of amastigote forms per infected cell[24]. Amphotericin B was used as the reference drug.

### Cytotoxicity against Mammalian Cells

Mouse peritoneal macrophages were used for cytotoxicity assay. The cells were incubated with compounds in a sixfold dilution from 30.0 to 2.0 µg/mL (for compounds **3**–**5**) and from 40.0 to 2.0 µg/mL (for compounds **6**–**11**), in triplicate at each concentration. The viability of the macrophages was determined with the MTT assay and was confirmed by comparing the control group morphology via light microscopy[21].

### Statistical Analysis

For promastigote forms of *Leishmania* assay and cytotoxicity on macrophages, the $IC_{50}$ values were carried out at 5% significance level ($p < 0.05$, CI 95%), calculated using a nonlinear regression curve, by using *GraFit* Version 5 software (Erithacus Software, Horley, U.K). For *Leishmania* amastigote assays, the statistical analysis was performed with the program GraphPad Prism 4 (GraphPad Software, San Diego, CA). One-way ANOVA was applied to compare all the groups. To compare the control with each compound, concentration was applied Dunnett post-test. Differences were regarded as significant when $p < 0.0001$ (***) and $p < 0.001$ (**).

## RESULTS AND DISCUSSION

In the present study, the imidazolidine derivatives were screened for their cytotoxicity against *Leishmania* and macrophages.

The antiproliferative activity of compounds **3–11** against *L. amazonensis* and *L. major* promastigote forms are described in Table 2. The results are expressed as the concentrations inhibiting parasite growth by 50% ($IC_{50}$). The ethylenediamine derivative **4**, with a free amino group, and compounds **5**, **6**, **7**, and **11** showed the best antiproliferative activities against *L. amazonensis* and *L. major*. Compound **4** showed the highest activity against the two *Leishmania* species. In this series, compound **9** also displayed good *in vitro* activity against *L. major* promastigote forms. In general, *L. major* promastigotes were the most sensitive to the compounds tested. Compounds **3**, **8**, and **10** did not show activity against promastigote forms of *Leishmania* species. Amphotericin B, used as the reference drug, showed $IC_{50}$ values of 0.4 and 0.3 µg/mL on *L. amazonensis* and *L. major* promastigote forms, respectively.

In this work, two *Leishmania* species were used and the results reflect differences in sensitivity of these parasites to the compounds tested. This fact is not surprising and previous *in vitro* studies have shown differences in sensitivity of *Leishmania* species in different classes of drugs[23,25,26].

For cytotoxicity on macrophages, none of the compounds tested were shown to be toxic to mammalian cells in the maximum concentration tested (Table 2).

The *L. amazonensis*–infected BALB/c macrophage model was assayed, aiming to confirm the activity of the imidazolidine derivatives against the intracellular stage of the parasite[1,2,4]. In this series, compounds **4, 5, 6, 7,** and **11** were selected because of their high activity against *Leishmania* promastigotes and their low toxicity against murine macrophages (Fig. 1). When the parasites were treated with the compounds, a significant dose-dependent decrease of intracellular amastigotes was observed. All compounds showed a significant effect against the amastigote forms of *L. amazonensis*. Compounds **4** and **5** showed the best activity on intracellular amastigotes, with an $IC_{50}$ value of 2.0 and 9.4 µg/mL, respectively. For compounds **4** and **5**, the survival index was calculated as 2.2 and 0.0 for 45

**TABLE 2**
**Effect of the Compounds 3–11 against *L. amazonensis* and *L. major* Promastigote Forms and Murine Macrophages**

| Compounds | $IC_{50}$ (µg/mL) | | |
|---|---|---|---|
| | *L. amazonensis*[a] | *L. major* | Peritoneal Macrophages[a] |
| **3** | >30.0 | >30.0 | >30.0 |
| **4** | 1.9 | 1.8 | >30.0 |
| **5** | 4.7 | 2.4 | >30.0 |
| **6** | 13.6 | 4.0 | >30.0 |
| **7** | 9.0 | 3.0 | >40.0 |
| **8** | >40.0 | >40.0 | >40.0 |
| **9** | >40.0 | 8.6 | >40.0 |
| **10** | >40.0 | >40.0 | >40.0 |
| **11** | 12.4 | 6.7 | >40.0 |

[a] Maximum concentration tested: 30.0 µg/mL for compounds **3–6** and 40.0 µg/mL for compounds **7–11**.

µg/mL, 12.6 and 2.7 for 30 µg/mL, 15.7 and 17.8 for 15 µg/mL, 16.6 and 132.78 for 7.5 µg/mL, and 300.0 and 323.5 for 1.5 µg/mL, respectively. These results correspond to an inhibition of survival index of 99.6, 98.0, 97.5, 97.0, and 55.0%, respectively, for compound **4,** and 100.0, 99.6, 97.0, 80.0, and 51.3%, respectively, for compound **5**. Amphotericin B, the reference drug, inhibited 63.1% of amastigotes in 5 µg/mL in 72 h after treatment.

### CONCLUSION

In summary, this work describes the synthesis and leishmanicidal activity of ethylenediamine and imidazolidine derivatives. The compounds showed a good activity against *Leishmania* without cytotoxicity on macrophages at the maximum concentration tested. Five compounds (**4–7** and **11**) displayed a good activity on promastigote forms of *Leishmania* species. Compounds **4** and **5** showed the best activity against *L. amazonensis* amastigote forms, indicated by the greater reduction in survival indices of parasites. In general, the addition of an aromatic substituent in position 2 of the imidazolidine ring decreased the antileishmanial activity. The biological activity shown by compound **4** coincides with the presence of the group ethylenediamine in the structure that interferes with the polyamine metabolism[5,6,7,8]. On the other hand, the less polar substitution in position 2 of the imidazolidine ring (compound **5**) suggests that the compound has a good cellular penetration across biological membranes.

### ACKNOWLEDGMENTS

The authors gratefully acknowledge UFJF, CAPES, and CNPq for fellowships. This research was supported by FAPEMIG.

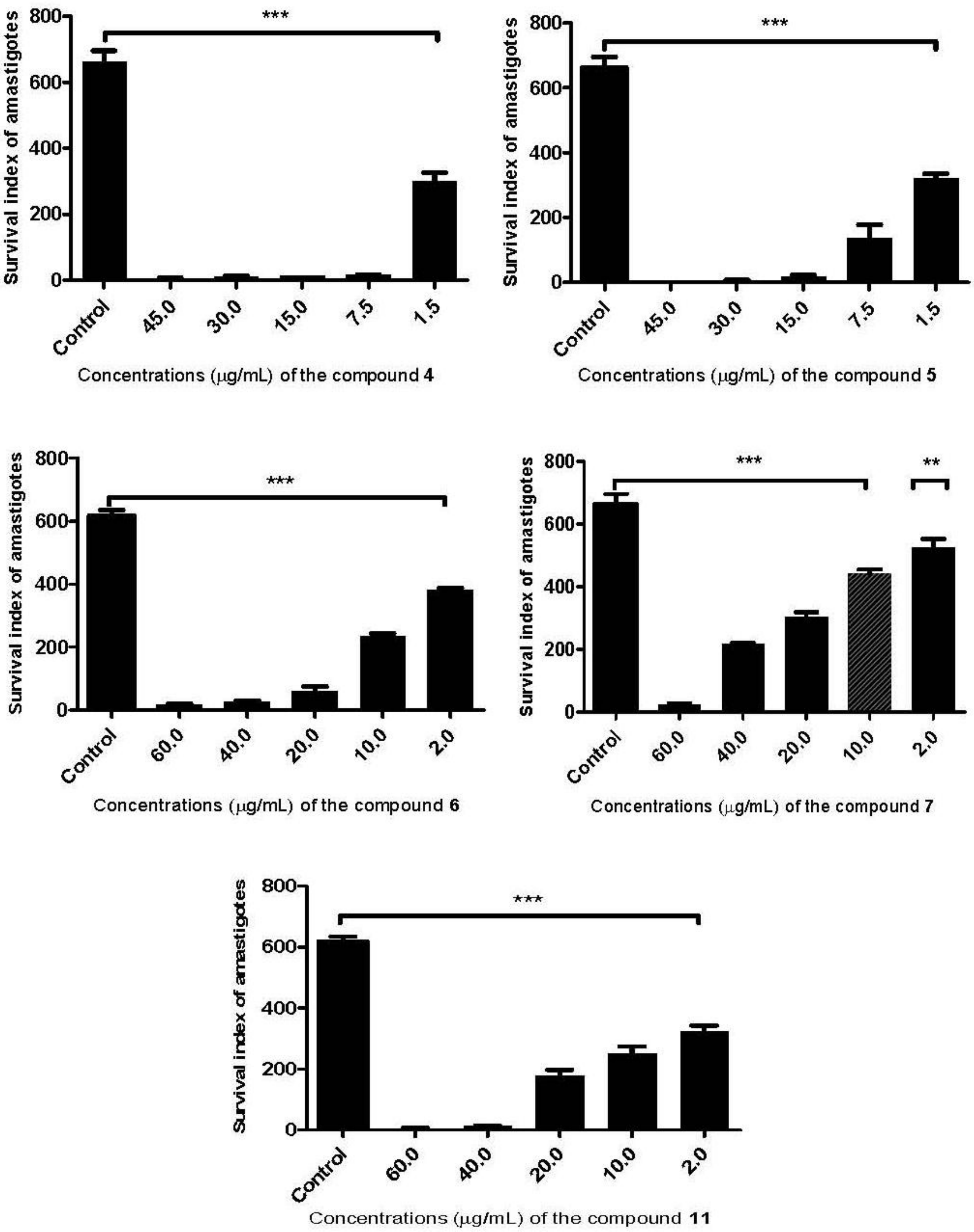

**FIGURE 1.** Effect of the imidazolidine derivatives on *L. amazonensis* interiorized in peritoneal macrophage cells. Statistically significant difference of control: **$p < 0.001$; *** $p < 0.0001$.
